# Expression of uPAR in tumor-associated stromal cells is associated with colorectal cancer patient prognosis: a TMA study

**DOI:** 10.1186/1471-2407-14-269

**Published:** 2014-04-17

**Authors:** Martin C Boonstra, Floris PR Verbeek, Andrew P Mazar, Hendrica AJM Prevoo, Peter JK Kuppen, Cornelis JH van de Velde, Alexander L Vahrmeijer, Cornelis FM Sier

**Affiliations:** 1 Department of Surgery, Leiden University Medical Center, Albinusdreef 2, 2300 RC Leiden, The Netherlands; 2 Chemistry of Life Processes Institute and Robert H. Lurie Comprehensive Cancer Center, Northwestern University, Evanston, IL, USA; 3 Antibodies for Research Applications BV, Gouda, The Netherlands

**Keywords:** Urokinase receptor, Immunohistochemistry, Diagnosis, Survival, Tumor associated stromal cell, Macrophage

## Abstract

**Background:**

The receptor for urokinase-type plasminogen activator (uPAR) is associated with cancer development and progression. Within the tumor microenvironment uPAR is expressed by malignant cells as well as tumor-associated stromal cells. However, the contribution of uPAR expression in these stromal cells to malignancy and patient survival in colorectal cancer is still unclear. This study compares the association of uPAR expression in both colorectal tumor-associated stromal cells and neoplastic cells with clinico-pathological characteristics and patient survival using tissue micro arrays (TMA).

**Methods:**

Immunohistochemical staining of uPAR expression was performed on tumor tissue from 262 colorectal cancer patients. Kaplan-Meier, log rank, and uni- and multivariate Cox’s regression analyses were used to calculate associations between uPAR expression and patient survival.

**Results:**

In the colorectal tumor-associated stromal microenvironment, uPAR is expressed in macrophages, (neoangiogenic) endothelial cells and myofibroblasts. uPAR expression in tumor-associated stromal cells and neoplastic cells (and both combined) were negatively associated with overall survival (OS) and Disease Free Survival (DFS). Uni- and multivariate Cox’s regression analysis for combined uPAR expression in tumor-associated stromal and neoplastic cells showed significant and independent negative associations with OS and DFS. Only uPAR expression in tumor-associated stromal cells showed independent significance in the uni- and multivariate analysis for DFS.

**Conclusion:**

This study demonstrates a significant independent negative association between colorectal cancer patient survival and uPAR expression in especially tumor-associated stromal cells.

## Background

In various cancer types, enhanced expression of uPAR is associated with worse patient prognosis and survival. This study evaluates in colorectal cancer whether the association is dependent on uPAR expression by malignant cells or on uPAR expression by tumor surrounding stromal cells. The results showed that high uPAR expression by malignant cells as well as high uPAR expression by tumor-associated stromal cells were independently correlated with worse patient survival.

### Introduction

Although the incidence of colorectal cancer is varying worldwide, in the western world it is the third most frequent cancer type [1]. To date, primarily the anatomic extent of the tumor is used to predict patient prognosis and select optimal treatment strategies. However these classification systems are rather unspecific. Novel techniques, able to define unique molecular tumor characteristics, would allow a more personalized approach to improve patient care. As a consequence, biomarkers that are able to classify tumors and are coherent with patient survival are desperately needed [2]. The urokinase-type plasminogen activator receptor (uPAR) might have the potential to be such a marker [3].

A pivotal characteristic of malignant tumors is the increased ability to degrade extracellular matrix (ECM), enabling malignant invasion and metastasis. The urokinase-type plasminogen activation (uPA) system plays a key role in tissue remodeling and ECM degrading [4]. uPAR, the membrane-bound receptor for uPA, has originally been identified in a monocyte/macrophage human cell line and is recognized in many physiological and pathologic conditions in which tissue remodeling is involved [5]. Binding of uPA to uPAR is a pre-requisite for the local activation of uPA, initiating plasmin-mediated extracellular matrix degrading [6]. Therefore, uPAR expression is closely related with pericellular proteolysis and in that manner facilitates (cancer) cell migration and invasion. Besides its receptor function, uPAR also mediates cell signaling, chemotaxis, proliferation, and tumor survival [7].

Over-expression of the urokinase receptor has been determined in the majority of malignant tumors, including pre-malignant colorectal adenocarcinomas, colorectal cancers and colorectal metastases [3,8,9]. Expression of uPAR is observed in both neoplastic as well as tumor-associated stromal cells of various tumor types including colorectal [10-12]. However, the correlation of uPAR expression in these stromal cells with malignancy and patient survival in colorectal cancer is still unclear. This study investigated the relationship of uPAR expression in tumor-associated stromal cell with clinical and follow-up data in a large panel of tumor tissues from colorectal cancer patients.

## Methods

### Patient and tumor characteristics

Tumor tissue samples were obtained from 262 patients in the period from 1991 to 2001 at time of primary surgery at the Leiden University Medical Center and were evaluated for histo-pathological characteristics by qualified pathologists according to current standards. Patient and tumor characteristics were collected retrospectively and are partly depicted in Table 1. Patients with pre-operative therapy or a history of cancer other than basal cell carcinoma (n = 9) or cervical carcinoma in situ (n = 1), and tumors which could not be evaluated for uPAR expression in both tumor-associated stromal cells and neoplastic cells were excluded, resulting in 262 usable tumors. Median age at operation was 66 years (range 30–91) and 136 (52%) patients were men. All patients had a proven primary adenocarcinoma of which 98 (37%) were located in the right colon, 99 (38%) in the left, and 65 (25%) in the rectum. Median follow-up was 7.7 years (range 0–20) calculated from the date of surgery. Tumor staging was determined using the tumor node metastasis (TNM) classification. Tumor differentiation grades were available for 207 patients; 49 (24%) tumors were well differentiated and 158 (76%) were moderate or poorly differentiated. Distant metastasis developed in 44 (17%) patients. At the end of the follow-up period 96 of the patients (37%) were still alive. All samples were handled in an anonymous fashion according to the national ethical guidelines (‘Code for Proper Secondary Use of Human Tissue,’ Dutch Federation of Medical Scientific Societies) and approved by the Institutional Ethics Committee of the Leiden University Medical Center.

**Table 1:** Uni- and multivariate analyses for Overall Survival (OS) and Disease Free Survival (DFS) on patient/tumor characteristics and uPAR expression in tumor-associated stromal cells and neoplastic cells (and both combined) from 262 colorectal tumor patients are displayed

			**Overall Survival (OS)**						**Disease Free Survival (DFS)**					
			**Univariate (OS)**			**Multivariate (OS)**			**Univariate (DFS)**			**Multivariate (DFS)**		
**Parameter**		**n**	**HR**	**95% CI**	**p-value**	**HR**	**95% CI**	**p-value**	**HR**	**95% CI**	**p-value**	**HR**	**95% CI**	**p-value**
Clinical data (total)		262												
Age in years	<65	109	1.000		**0.000**	1.000		**0.000**	1.000		**0.000**	1.000		**0.000**
	≥65	153	3.381	2.360-4.842		3.648	2.537-5.245		3.058	2.172-4.307		3.309	2.338-4.682	
TNM	Stage I	47	1.000		**0.000**	1.000		**0.000**	1.000		**0.000**	1.000		**0.000**
	Stage II	97	1.522	0.910-2.547		1.699	1.013-2.850		1.513	0.927-2.469		1.686	1.030-2.760	
	Stage III/IV	118	3.590	2.208-5.838		4.034	2.473-6.580		3.199	2.006-5.102		3.560	2.223-5.700	
Differentiation^I^	Grade 1	49	1.000		0.451				1.000		0.663			
	Grade 2+	158	1.174	0.773-1.783					1.093	0.733-1.630				
MSI^II^	MSS	184	1.000		0.457				1.000		0.429			
	MSI	26	0.816	0.477-1.396					0.815	0.491-1.353				
Size^III^ in mm	<50	179	1.000		0.589				1.000		0.396			
	≥50	82	1.093	0.791-1.511					1.146	0.837-1.568				
Mucinous^IV^	No	220	1.000		0.447				1.000		0.248			
	yes	36	0.850	0.558-1.293					0.790	0.529-1.179				
uPAR in tumor cells	Low	152	1.000		**0.036**	1.000		0.177	1.000		**0.037**	1.000		0.318
	High	110	1.387	1.021-1.883		1.241	0.907-1.697		1.372	1.019-1.848		1.168	0.861-1.585	
uPAR in stromal cells	Neg	38	1.000		**0.033**	1.000		0.067	1.000		**0.014**	1.000		**0.031**
	Pos	224	1.688	1.044-2.729		1.580	0.968-2.578		1.819	1.127-2.936		1.713	1.049-2.796	
Combined uPAR expression^V^	Neg	38	1.000		**0.032**	1.000		**0.037**	1.000		**0.019**	1.000		**0.032**
	Pos/Low	119	1.508	0.909-2.501		1.503	0.905-2.495		1.646	0.995-2.724		1.661	1.002-2.754	
	Pos/High	105	1.925	1.159-3.198		1.906	1.146-3.170		2.036	1.230-3.370		1.959	1.182-3.247	

P-values of 0.05 or less are considered significant and are presented in bold. Both uPAR expressions in neoplastic and tumor-associated stromal cells were significantly related to OS in the univariate analysis. Both neoplastic and tumor-associated stromal cell uPAR expression showed significance in the univariate analysis for DFS. Combined uPAR expression reached significance for OS and DFS in a multivariate Cox’s regression analysis including the factors age and TNM which were significant in the univariate analysis.

^I^207 patients, ^II^210 patients, ^III^261 patients, ^IV^256 patients, ^V^Multivariate analysis is performed with combined uPAR expression.

### Tissue micro array (TMA) production

Formalin-fixed paraffin-embedded tissue blocks of the primary tumors were collected from the pathology department. Sections were cut for haematoxylin-eosin staining and histopathologically representative tumor regions were used for preparation of TMA blocks. From each donor block, three 0.6 mm diameter tissue cores were punched from tumor areas and transferred into a recipient paraffin block using a custom-made precision instrument. Because the TMA was designed to evaluate protein expression throughout the whole tumor, cores where taken from three different locations across the tumor tissue, avoiding necrotic or invasive areas.

### Immunohistochemistry

Immunohistochemical (IHC) staining on the TMA was performed on 4 μm sections cut from each TMA receiver block. TMA sections were deparaffinized and rehydrated. Endogenous peroxidase was blocked for 20 minutes in 0.3% hydrogen peroxide in methanol. The slides were treated for antigen retrieval in citrate buffer for 10 minutes at 95°C (DAKO PT Link). Sections were incubated overnight with the uPAR specific antibodies at pre-determined optimal dilution. After 30 minutes of incubation with DAKO envision + HRP anti-mouse (K4001; DAKO Cytomation, Glostrup, Denmark) the sections were visualized using diaminobenzidine solution (DAB+; DAKO kit). Sections were counterstained with haematoxylin, dehydrated and finally mounted in malinol (Waldeck-division Chroma). To compensate for possible loss of antigen detectability due to the long inclusion period of the patients, the primary uPAR antibodies were incubated overnight [13]. The uPAR staining was not correlated with operation date (not shown).

IHC on the whole tumor slides was performed on 7 consecutive slides from the same tumor sample. Tissue sections from whole tumors were obtained and (pre) treated in the same manner as the TMA slides (described above) except for the section thickness (6 μm) and the mounting of the slides in Pertex (Histolab) instead of malinol. The consecutive tumor sections were simultaneously stained for the stromal markers. Antibodies against the following antigens in the corresponding concentrations were used: Vimentin for mesenchymal cells in 0.4 μg/ml (Santa Cruz; clone V9, Santa Cruz, USA), CD68 as marker for monocytes/macrophages in 2.5 μg/ml (DAKO; clone KP1), CD31 for endothelial cells in 1.7 μg/ml (DAKO; clone JC70A), CD105 for activated endothelial cells in 5 μg/ml (neo-angiogenic) (R&D systems, Abington, UK), α-SMA for myofibroblasts in 0.07 μg/ml (Progen; clone ASM-1, Heidelberg, Germany), cytokeratin for epithelial cells 0.4 μg/ml (DAKO; AE1/AE3) and uPAR/CD87 expression in 2.4 μg/ml (ATN-615) gift from prof. Mazar [14].

### Scoring methods

The 0.6 mm cores of all 262 colorectal cancer patients were semi-quantitatively scored for the proportion of uPAR positive neoplastic and tumor-associated stromal cells by two independent examiners (MB, FV). Cores were used when 50% or more was occupied by tissue. Patients with less than two evaluable cores were excluded which resulted in 182 (69.5%) patients with 3 evaluable cores and 80 (30.5%) patients with 2 usable cores. The percentage of positive tumor cells and stromal cells within each core were scored independently and categorized in 0–5, 5–25, 25–50, 50–75 and 75-100%. The median of the triplicate or duplicate cores were used for data analysis. In a preliminary log-rank/Kaplan-Meier analysis the discriminative values for uPAR scoring categories for Overall Survival (OS) and Disease Free Survival (DFS) were assessed (data not shown). In the final analysis percentages of uPAR staining were dichotomized as follows: absence (<5%) or presence (≥5%) of uPAR in tumor-associated stromal cells and low (<50%) or high (≥50%) expression of uPAR in neoplastic cells. The Spearman rank analysis and Kappa statistics were performed to calculate inter-observer agreement. To finalize the scoring, in case of discrepancies, both examiners reviewed the cores together to reach consensus.

### Statistical analysis

Statistical analyses were conducted using SPSS statistical software (version 20.0 for Windows, SPSS Inc, Chicago, USA). The Pearson Chi-Square test was performed to compare nominal variables. The Kaplan–Meier method was used for survival plotting and log-rank test for comparison of the survival curves. Time to events in OS and DFS analysis was defined as follows: from time of primary surgery to time of death or cancer relapse. Multivariate Cox’s proportional hazard analyses were performed with the factors that were significant in the univariate analysis, including age and TNM. Hazard ratios (HR) and their 95% confidence intervals (95% CI) are included. All statistical tests are conducted two-sided and p-values of 0.05 or less are considered significant.

## Results

### Patient and tumor characteristics

**Figure 1:**
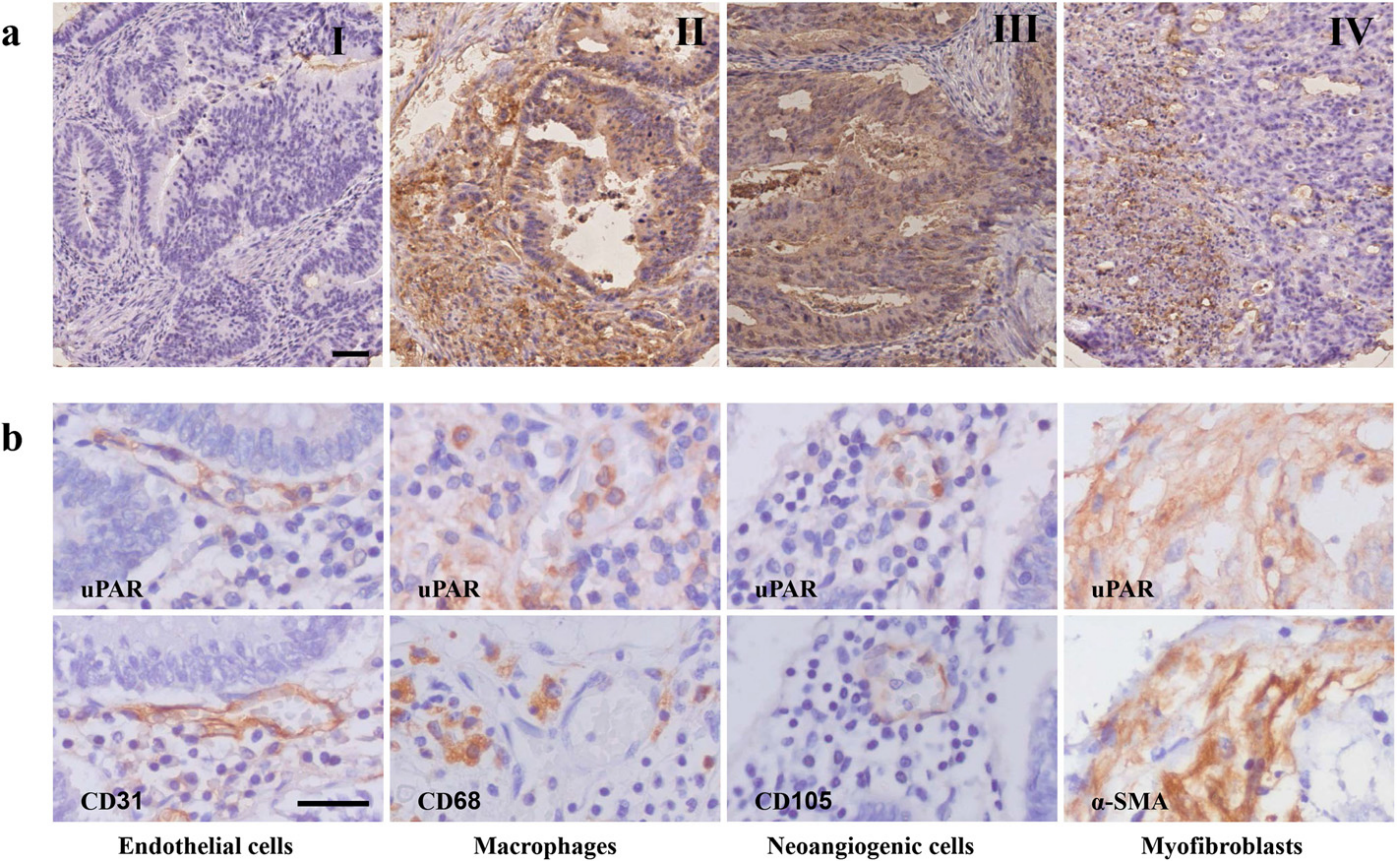
**Immunohistochemical stainings of colorectal cancer tissue sections with anti-uPAR and anti-cell marker antibodies. a)** uPAR staining on TMA cores from tissue of colorectal cancer patients: I) Negative in neoplastic and stromal cells, II) positive in neoplastic and stromal cells, III) positive in neoplastic and negative for stromal cells and IV) negative in neoplastic and positive for stromal cells. **b)** Sequential stained sections showing expression of the urokinase receptor in comparison with various markers for tumor-associated stromal cells: I) with endothelial cells, II) with monocytes/macrophages, III) neoangiogenic cells and IV) with myofibroblasts. Bars in a) and b) indicate ~50 μm.

Immunopositivity for uPAR in tumor-associated stromal cells was found in 224 (85%) tumors, whereas low uPAR expression in neoplastic cells was shown in 152 (58%) tumors. From all the clinico-pathological parameters, including the administration of adjuvant therapy and the grade, location, and size of the tumor, only high uPAR expression on tumor cells was significantly correlated, where high uPAR expression was found in well differentiated tumors. Further, no significant associations between uPAR expression and the clinico-pathological parameters were observed (data not shown). Inter- and intratumoral uPAR specific staining was variable and associated with both the cell membrane and cytoplasm in tumor and tumor-associated stromal cells (Figure 1a). The degree of expression was graded as low (58%) or high (42%) for uPAR immunoreactivity in neoplastic cells and negative (15%) or positive (85%) for expression in tumor-associated stromal cells. No correlation was found between uPAR expression on neoplastic cells and tumor-associated stromal cells (p = 0.063). A moderate agreement between observers was seen. For tumor cells, Spearman rank analysis gave 0.469 (p < 0.000) whereas Kappa statistics showed 0.47 (95% CI: 0.35-0.60). For stromal cells Spearman rank analysis gave 0.450 (p < 0.000) whereas Kappa statistics showed 0.42 (95% CI: 0.28-0.55).

### Survival analysis

**Figure 2:**
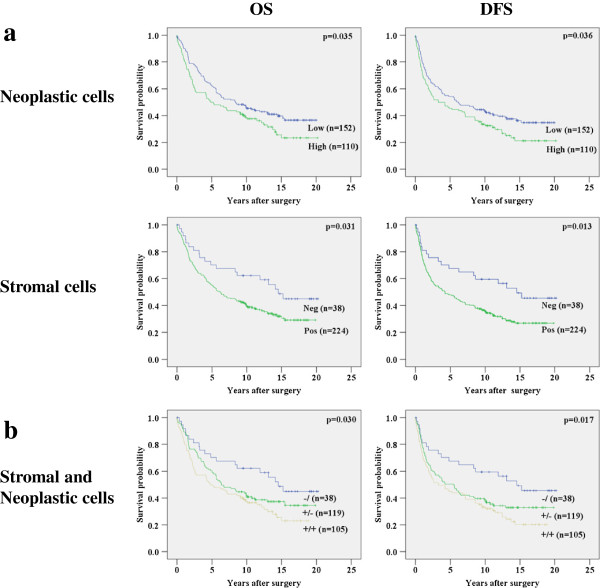
**Kaplan-Meier Overall Survival (OS) and Disease Free Survival (DFS) curves for uPAR expression in tumor tissues from 262 patients with colorectal cancer. a)** Neoplastic cells (low <50% & high ≥50%) and tumor-associated stromal cells (neg <5%, pos ≥5%). **b)** Combined uPAR expression, 3 groups: stromal cells negative for uPAR(−/), stromal cells positive for uPAR and low uPAR expression in neoplastic cells (+/−), stromal cells positive for uPAR and high expression of uPAR in neoplastic cells (+/+).

Kaplan Meier curves showed a significant negative association between uPAR expression in neoplastic and tumor-associated stromal cells with OS (p = 0.035 & p = 0.031) and DFS (p = 0.036 & p = 0.013, Figure 2a). Both uPAR expression in neoplastic and tumor-associated stromal cells were significant related to OS in the univariate analysis, but both did not retain significance in the multivariate analysis (p = 0.177 and p = 0.067, Table 1). For DFS, both neoplastic and tumor-associated stromal cell uPAR expression showed significance in the univariate analysis (p = 0.037 and p = 0.014), but only uPAR expression in tumor-associated stromal cells stayed significant in the multivariate analysis (p = 0.031).

The combination of uPAR expression in both tumor-associated stromal cells as well as neoplastic cells in three subgroups showed a stepwise correlation in the log rank analysis for both OS and DFS (p = 0.030 and p = 0.017) (Figure 2b). Patients with negative uPAR expression in tumor-associated stromal cells (−) showed better OS and DFS, whereas patients with uPAR expression in tumor-associated stromal cells as well as in neoplastic cells had the worst (+/+), with the rest group (+/−) in between. Combined uPAR expression reached significance for OS (p = 0.037) and DFS (p = 0.032) in the multivariate Cox’s regression analysis as shown in Table 1.

### uPAR expression in tumor-associated stromal cells

uPAR expression was present in various types of tumor-associated stromal cells in sequential sections from whole tumors. Figure 1b displays urokinase receptor staining in (neoangiogenic) endothelial cells, tumor-associated macrophages, and cancer-associated myofibroblasts. Not all monocytes/macrophages found on the slides expressed uPAR, but when present, it showed a more intense staining in comparison with uPAR positive neoplastic cells. uPAR expression was frequently observed in endothelial cells expressing both CD31 and CD105, underscoring the presence of the urokinase receptor in intra-tumoral neoangiogenic cells. uPAR was regularly but not consistently found in tumor-associated myofibroblasts. uPAR expression was especially associated with myofibroblasts located in the invasive front of the tumor.

## Discussion

This study demonstrates the relationship between enhanced expression of the urokinase receptor in colorectal tumor-associated stromal cells and a significant worse patient survival. Neoplastic cell and tumor-associated stromal cell expression of uPAR in colorectal cancers appeared to be independent from each other and patients with enhanced uPAR in both cell types showed the worst prognosis.

These data could partly explain the relatively strong correlation between uPAR and survival as found in especially breast cancer homogenate studies using ELISAs, versus the generally less strong associations noticed in immunohistochemical studies scoring specific tumor cell uPAR staining [3,15]. Immunohistochemical studies in general score uPAR in malignant cells, neglecting the stromal cells, whereas in ELISA-based studies the overall presence of uPAR is measured. Although the correlation between uPAR expression in neoplastic and tumor-associated stromal cells and patient prognosis has been studied before in breast cancer, the results are not consistent. Some studies showed no significant relation with prognosis [16,17], whereas others found a significant association with disease free survival or relapse-free survival but not with overall survival [18]. These variable results could partly be explained by the different IHC antibodies that were used. uPAR is present in diverse configurations which are not all detected equally well by the various anti-uPAR antibodies. To circumvent this, we used an antibody, ATN-615, which binds with high affinity (kd ~1 nM) to domain D3 of uPAR and is extensively validated [17]. Therefore, virtually all forms, i.e. full size or D2D3 fragments of cell-bound uPAR are detected regardless whether the ligand (uPA) is bound or not [19].

Our results obtained with TMA sections indicate that neoplastic cell positivity correlates with patient survival, like has been observed before using immunohistochemistry on whole tumor sections [20]. The use of TMA sections to differentiate between different locations of tumor markers within the tumor has been debated. To overcome the drawback of heterogeneity within the tumor, it is argued that increasing the number of cores per tumor will be sufficient [21]. Examining punches from different representative locations within the tumor may reveal an overview of the expression of an antigen similar to what is obtained from whole sections. So far, no consensus exists about the number and the location of the cores, which could also depend on the nature of the marker of interest. Our TMA consisted of 3 punches from different representative locations within the tumor.

Several scoring methods are used for the evaluation of immunohistochemical stainings. We used only the proportion of neoplastic and tumor stromal cells which express uPAR rather than the combination with the intensity of the stainings like for instance the score proposed by Remmele [22].

A recent study in gastric tumors indicates that especially uPAR expression in gastric cancer cells in the peripheral invasion zone is an independent prognostic factor for overall survival [23]. Our observations in colorectal cancer support these findings. In the colorectal tumor-associated stromal microenvironment, uPAR expression was furthermore observed in monocytes/macrophages, (neoangiogenic) endothelial cells and myofibroblasts, which is in line with previously published studies [8,16,24]. Macrophages and myofibroblasts are able to induce neoplastic tumor cell proliferation, progression and metastasis via the secretion of growth factors and cytokines [25-27]. Myofibroblasts located in the tumor microenvironment modulate inflammatory responses by secreting pro-inflammatory cytokines, resulting in recruitment of immune cells such as macrophages. Tumor-associated macrophages induce several tumor promoting processes such as angiogenesis, extracellular matrix breakdown, tumor cell migration, invasion, and metastasis [28]. The pro-angiogenic growth factors secreted by both the tumor-associated myofibroblasts and macrophages contribute to the ‘angiogenic switch’. This switch results in vasculogenesis and the recruitment of existing endothelial cells to proliferate, migrate, and form new blood vessels which offer the tumor the ability to secure a steady supply of oxygen and nutrients and to metastasize [29]. Overexpression of uPAR, focusing the local proteolytic activity which is essential for matrix remodeling, seems to be a common feature, not only for invasive cells, but also for cells that play key roles in tumor cell support. The association between uPAR expression in the cancer-associated-stromal-cells and the survival of the patients is probably a direct consequence of the supportive effects of these cells on tumor proliferation. Our finding that stromal cells contribute substantially to the overall uPAR content within colorectal cancers could also have an impact on the prognostic relevance of soluble uPAR (suPAR) as has been reported by a number of studies for various cancer types [30-35]. In general, enhanced levels of s-uPAR and s-uPAR fragments in blood and urine are found to be related with poor prognosis. But the pattern of secreted suPAR-fragments was highly diverse in a small number of ovarian carcinoma patients, which could have been caused by the of cell population within the individual tumors [36].

The multi-faceted appearance of uPAR might also implicate a potential role as tumor target. The possibility to target multiple relevant cell types within the same tumor might compensate for the relatively low overexpression on malignant cancer cells compared with other tumor markers, like EGFR, Her2/Neu or EpCAM. Using the same anti-uPAR antibody as used in this study, tumor regression was achieved *in vivo* in mice xenografted with ovarian, colon and prostate cancer [37-39].

## Conclusions

uPAR plays a major role in adhesion, migration, invasion and metastasis of cancer. It is found in the majority of colorectal tumors in malignant cells and in various types of tumor-supportive tumor-associated stromal cells. Although this study does not especially discriminate between the different stromal cells, our results show a significant independent association between colorectal cancer patient survival and uPAR expression in the general tumor-associated stromal cells.

## Abbreviations

uPAR: urokinase-type Plasminogen Activator Receptor; suPAR: soluble urokinase-type Plasminogen Activator Receptor; uPA: urokinase-type Plasminogen Activator; ECM: Extracellular matrix; TNM: Tumor nodes metastasis; HR: Hazard ratio; OS: Overall survival; DFS: Disease free survival; TMA: Tissue microarray; IHC: Immunohistochemistry; MSI: Microsatellite instability; MSS: Microsatellite stable.

## Competing interests

A.P. Mazar is employed as a Managing Member in Tactic Pharma and has ownership interest (including patents) in the same. No potential conflicts of interest were disclosed by the other authors.

## Authors’ contributions

MB designed and coordinated the overall study together with FV, PK, AV and CS. MB and FV were involved in TMA scoring, pathology review, data analysis, scoring of stains and drafting and finalizing the manuscript. AM helped with the interpretation of the results, drafted the manuscript and revised it critically for important intellectual content. HP performed IHC, assisted in histo-pathological analysis and helped with editing and finalizing the manuscript. PK contributed to the design of the study, interpretation of the results as well as preparation and finalizing of the manuscript. CvdV assisted in study design, contributed to the manuscript writing and revised it critically for important intellectual content. AV co-designed the overall study, contributed to the manuscript writing and revised it critically for important intellectual content. CS co-designed and coordinated the overall study. He was involved in data analysis and drafted, finalized and critically revised the manuscript. All authors read and approved the final manuscript.

## Pre-publication history

The pre-publication history for this paper can be accessed here:

http://www.biomedcentral.com/1471-2407/14/269/prepub
